# Maternal verbal aggression in early infancy and child’s internalizing symptoms: interaction by common oxytocin polymorphisms

**DOI:** 10.1007/s00406-019-01013-0

**Published:** 2019-05-07

**Authors:** Laetitia J. C. A. Smarius, Thea G. A. Strieder, Theo A. H. Doreleijers, Tanja G. M. Vrijkotte, M. Hadi Zafarmand, Susanne R. de Rooij

**Affiliations:** 1 Department of Public Health, Amsterdam Public Health Research Institute, Academic UMC, University of Amsterdam, PO Box 22660, 1100 DD Amsterdam, The Netherlands; 2 Academic Center for Child and Adolescent Psychiatry de Bascule, Amsterdam, The Netherlands; 3 Department of Child and Adolescent Psychiatry, Amsterdam UMC, location VU University Medical Center, Amsterdam, The Netherlands; 4 Arkin Institute for Mental Health, Amsterdam, The Netherlands; 5 Department of Clinical Epidemiology, Biostatistics and Bio-informatics, Amsterdam UMC, University of Amsterdam, Amsterdam, The Netherlands

**Keywords:** Verbal abuse, rs53576, rs2268498, rs2740210, rs4813627, Infant

## Abstract

Genetic predisposition of social sensitivity might affect vulnerability to develop psychopathology after early life stress exposure. This study examined whether maternal verbally aggressive behavior in early infancy interacts with oxytocin polymorphisms in developing internalizing symptoms at ages 5–6 and 11–12. In the Amsterdam-Born-Children-and-their-Development (ABCD) study, a large observational, population-based birth cohort, maternal verbally aggressive behavior was assessed in the 13th postnatal week by a self-report questionnaire. Internalizing symptoms at age 5–6 were assessed by maternal report (*N* = 969) and internalizing symptoms at age 11–12 were assessed by self-report (*N* = 750). Data on oxytocin receptor polymorphisms rs53576 and rs2268498 and oxytocin polymorphisms rs2740210 and rs4813627 were collected. If the child was carrier of rs2740210 CA/AA polymorphism, exposure to maternal verbally aggressive behavior (10.6%) was positively associated with general anxiety at age 5–6 and emotional symptoms at age 11–12 (*p* for interaction = 0.011 and *p* = 0.015, respectively). If the child was carrier of rs4813627 GG (wild type), exposure to maternal verbally aggressive behavior was negatively associated with anxiety sensitivity and emotional symptoms at age 11–12 (*p* for interaction = 0.011 and *p* = 0.022, respectively). After exposure to maternal verbally aggressive behavior in early infancy, oxytocin polymorphisms may partly determine a child’s vulnerability to internalizing symptoms.

**Electronic supplementary material:**

The online version of this article (10.1007/s00406-019-01013-0) contains supplementary material, which is available to authorized users.

## Introduction

Since vocalizations may be as important as touch to the neuroendocrine regulation of social bonding [1], maternal verbally aggressive behavior can be considered stressful to the infant. The developing brain is most vulnerable for environmental influences at periods of rapid growth and development, such as early infancy. The striatum, part of the dopamine, serotonin, glucocorticoid, GABA-nergic, and affiliated oxytocin pathways, can be considered as a central entry port for processing emotional/motivational information [2]. The striatum and its massive projections from the frontal cortex, amygdala ,and hippocampus [3, 4] are involved in detecting attachment-relevant cues, in appraising their valence, and in guiding action by coding the affective attributes of stimuli [5, 6]. Synaptogenesis in the striatum is most rapid between 2–4 months of age and total gray matter volume reaches adult size at about 4 months of age [7], both of which implicate the relevance of stress in this period of life. The striatum consists of the ventral striatum (nucleus accumbens and olfactory tubercle) and dorsal striatum (caudate nucleus and putamen) and is activated by unexpected or intense stimuli [8]. The impact of maternal verbally aggressive behavior on child development during this early infancy period could be considerable.

Retrospective evidence shows that verbal abuse during childhood has been associated with various psychiatric disorders in adulthood: mood and anxiety disorders, eating disorders, substance abuse disorders, personality disorders, and schizophrenia [9]. Moreover, in a study of 9–12 year old, any level of maternal verbal aggression greater than one or two instances per year has been associated with depressive symptoms, delinquency, peer overt and relational victimization, and low self-esteem in pre-adolescence [10]. Importantly, parental threatening, hostile, and rejecting behaviors have been shown to predict overall anxiety sensitivity (AS) [11], which is an important contributor in the association between abuse and the development of borderline personality disorder symptoms [12]. Verbal abuse may particularly influence risk for internalizing disorders, as verbal abuse influences the development of a self-critical style [13].

Although verbal abuse is an important aetiopathogenic factor in the development of several psychiatric phenotypes, children are affected in various ways; some children even show remarkable resilience. Differences in vulnerability or resilience are related to protective environmental factors during development, including parental or non-parental support, and biological inborn differences in genetic profile [14]. The extent to which maternal verbally aggressive behavior in infancy is experienced as stressful and hereby potentially affects internalizing symptoms might partly depend on infant’s genetic make-up. Candidates for gene-environment interaction would be a number of single-nucleotide polymorphisms (SNPs) in the oxytocin (OXT) gene and oxytocin receptor (OXTR) gene, due to their differential associations with social sensitivity.

G-allele carriers of rs53576, located in the third intron of the OXTR gene, have been associated with increased empathic abilities [15]. Recently, the rs53576 G-allele has been associated with increased amygdala responsiveness to emotional facial expressions [16], compared to the A-allele, implying differences in arousal depending on OXTR variant. Of note, evidence of gene-environment interaction of early life stress and rs53576 is contradictory and solely based on retrospective recall. After several kinds of maltreatment in childhood (emotional, physical or sexual abuse; emotional or physical neglect), G-allele carriers have shown increased depressive symptoms [17, 18], and conduct problems (in females only) [19] and GG carriers showed a higher risk of emotional dysregulation [20], compared to A-carriers. However, a study on adults with clinically diagnosed depression and anxiety disorders showed no interaction of rs53576 with one or more types of childhood maltreatment [21]. Another candidate OXTR polymorphism is rs2268498. Compared to those carrying C alleles, T-allele (TT/TC) carriers have been demonstrated to have better recognition of facial emotion [22] and self-reported empathy [23, 24]. In addition, two specific OXT polymorphisms, rs2740210, and rs4813627, located in an intron, have been found associated with maternal infant-directed vocalizing (duration) or exaggerated prosodic cues [25]. It is unclear which infant’s phenotype is associated with these OXT polymorphisms, because their phenotypes have only been investigated in mothers. Although speculative, it is plausible that rs2740210 and rs4813627 are not only related to social functions in the mothers, but to social functions in the infants as well. In the present study, it is hypothesized that infants might experience maternal verbally aggressive behavior differently depending on OXTR or OXT polymorphisms. We specifically expected that infants carrying OXTR rs53576 GG or rs2268498 TT/TC variant, who are more apt at recognizing facial emotions, experience more stress in response to maternal verbally aggressive behavior and display more internalizing symptoms both at age 5–6 and at age 11–12, compared to infants carrying, respectively, GA/AA or CC variant. We also explored whether exposed infants carrying one of the different variants of OXT rs2740210 or rs4813627 were more or less vulnerable to internalizing symptoms, compared to exposed infants carrying the other variant.

## Materials and methods

### Participants

The study sample is part of a large prospective, observational, population-based multiethnic birth cohort, the Amsterdam Born Children and their Development (ABCD) study, which started in 2003. Extensive information about the cohort and procedures regarding data collection has been published elsewhere [26]. Approval of the study was obtained from the Central Committee on Research involving Human Subjects in The Netherlands, the Medical Ethical Committees of participating hospitals, and the Registration Committee of the Municipality of Amsterdam. Approval of the ABCD-Genetic Enrichment (ABCD-GE) study, a sub-study of 1192 white children, was obtained from the Medical Ethical Committees of the Academic Medical Centre in Amsterdam. For the ABCD-GE study, an opt out procedure was followed. Written informed consent was obtained from all participating mothers and from children aged 12 or older.

**Fig. 1:**
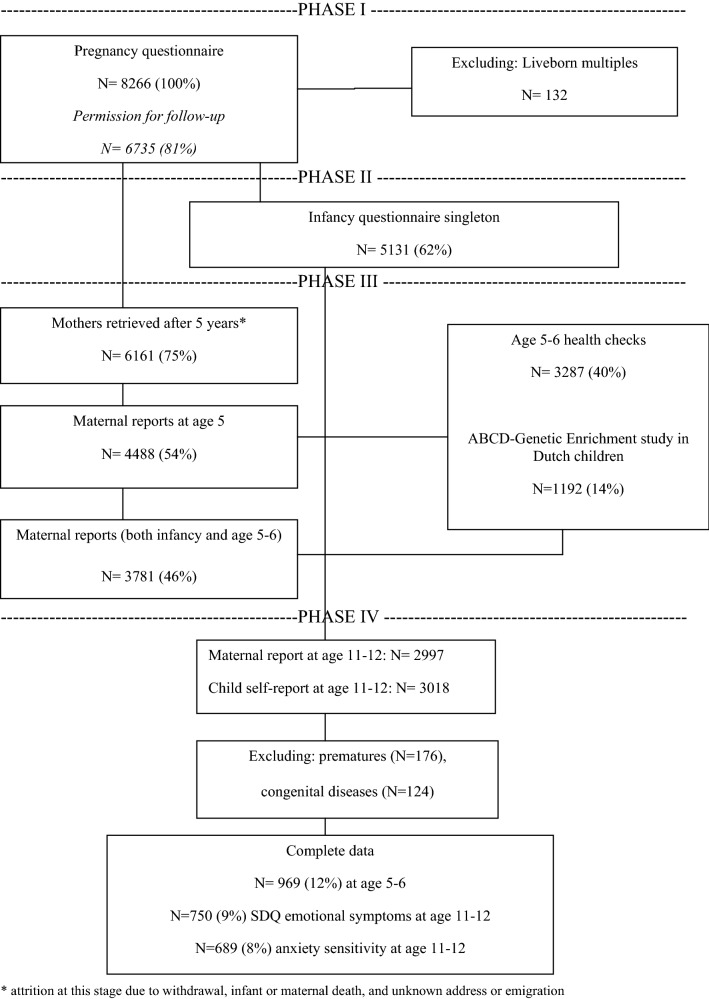
Flow chart of participants included for analysis; * attrition at this stage due to withdrawal, infant or maternal death, and unknown address or emigration

Between January 2003 and March 2004, all pregnant women living in Amsterdam were asked to participate in the ABCD study during their first prenatal visit to an obstetric care provider. Figure 1 shows a flow chart of characteristics of the participants included in the present study. Multiple births were excluded from the cohort preceding the third phase. For the ABCD-GE study, blood of the child was collected from a simple finger prick during the 5-year health check-up. DNA was extracted from the dried blood spots. Data on the first three measurements (pregnancy, infancy, and early childhood) had to be available to be included in the present study. We excluded preterm births (gestational age < 37 weeks) and congenital diseases (*N* = 969 included). At age 11–12, 689 children and mothers were included in anxiety sensitivity analyses and 750 in SDQ emotional symptoms analyses.

### Maternal verbally aggressive behavior in infancy

Maternal verbally aggressive behavior was assessed by maternal self-report completed in the 13th week after birth (mean 13th week, range 11–25 weeks, and SD 2 weeks), by a question on frequency of speaking angrily on a six point scale: ‘Have you ever spoken angrily to your baby to diminish the crying?’ The score was dichotomized [speaking angrily (frequency ≤ 1 or ≥ 2)]. Maternal verbally aggressive behavior was considered present if speaking angrily had been present at least twice [27].

### Internalizing symptoms at age 5–6 and at age 11–12

Emotional symptoms were assessed by maternal report at age 5–6 and by self-report at age 11–12, using the emotional symptoms subscale of the Dutch translation of Goodman’s Strengths and Difficulties Questionnaire (SDQ) [28], a short-screening questionnaire suitable for 4–16 years. Children’s general anxiety at age 5–6 was assessed by maternal report, using the generalized anxiety subscale of the validated Preschool Anxiety Scale (PAS) [29]. Pre-adolescent anxiety sensitivity was assessed by self-report at age 11–12, using the anxiety sensitivity subscale of the Substance Use Risk Profile Scale (SURPS) [30].

### Genotyping

DNA samples were genotyped using the Illumina Human Core Exom Beadchip (Illumina, San Diego, CA, USA), which includes over 540,000 genetic markers. Before imputation, SNPs were excluded if they had high levels of missing data (SNP call rate < 95%), strong departures from Hardy–Weinberg equilibrium (*p* < 1 × 10^−6^), or low Minor allele frequencies (MAF) ( < 1%). Individuals were excluded if mismatch in heterozygosity, gender, or relatedness existed. Genetic markers were imputed using the IMPUTE2 software and the 1000 Genomes References Panel (phase 1 release v3, build 37). Genotypes for the SNPs of interest were extracted from the imputed genome-wide association study (GWAS) data set. The final number of SNPs was 277,644. The total of SNPs after imputation was 27,448,454. We performed post-imputation quality control and removed monomorphic variants (MAF = 0), as well as variants which were extremely rare in the cohort (MAF < 1%). Using the IMPUTE2-INFO metrics, the commonly used > 0.4 threshold [31] was efficient to discriminate between well and poorly inferred genotypes at the MAF ≥ 1 in our sample. Based on literature on sample size, statistics, and selection of SNPs in genetic epidemiologic studies [32–35], a maximum of four SNPs were selected. These SNPs have previously been associated with either recognition of facial emotions or vocalizing and have a MAF > 0.25, hereby suiting the power of our study. A carrier model was chosen to compare the variants differentially known to be associated with recognition of facial emotions [OXTR *rs53576* (GG vs. GA/AA) or *rs2268498* (TT/TC vs. CC)] or duration of vocalizing (OXT *rs2740210* (CC vs. CA/AA) or *rs4813627* (GG vs. GA/AA). The mean quality of the imputed genotypes (*r*^2^) of the 4 SNPs included in this study (rs53576, rs2268498, rs2740210, and rs4813627) was 0.82, ranging from 0.75 to 0.87 (Table 1). Using a whole genome association analysis Java-based toolset (PLINK), none of the selected SNPs were in pairwise linkage disequilibrium (*R*^2^ = 0.31 and 0.28 for rs53576/rs2268498 and rs2740210/rs4813627, respectively).

**Table 1:** Oxytocin polymorphism variants and imputation quality

Chr	rs_id	Bp position	Minor/major allele	MAF	Coded allele	Quality of imputation	Certainty	HWE *p* value
3	rs53576	8804371	A/G	0.35	G	0.87	0.925	0.52
3	rs2268498	8812411	C/T	0.45	C	0.87	0.916	0.91
20	rs2740210	3053255	A/C	0.30	A	0.75	0.866	0.73
20	rs4813627	3055513	A/G	0.50	G	0.77	0.851	0.19

*MAF* minor allele frequency, *HWE *Hardy–Weinberg equilibrium

### Covariates

Infant characteristics included sex, birth weight, gestational age, and excessive crying [36]. The following maternal characteristics were assessed during pregnancy: age, parity (0 or ≥ 1), cohabitation status (single or living with partner), and level of education (years after primary school). During infancy, maternal smoking at home (yes or no), maternal depressive symptoms (Center for Epidemiologic Studies Depression Scale (CES-D), total score [37], and maternal physical aggressive behavior [27] were included. Pleasure in infant care was measured using five questions on a four point scale [27].

Covariates at age 5 included: authoritarian parenting style [12-items subscale of the short version of the Parenting Styles and Dimensions Questionnaire (PSDQ)] [38, 39], maternal parenting stress (9 items on attachment derived from the 123 items of the ‘Nijmeegse Ouderlijke Stress Index’ (NOSI-K) [40] and maternal depressive symptoms (depression severity subscale of the Depression Anxiety Stress Scales (DASS 21) [41], all of which were analyzed continuously. Whether the child was being raised in an intact family (a family in which the child lives with both biological parents in the same house: yes or no) was assessed by one item, both at age 5–6 and age 11–12. Covariates at age 11–12 included: Tanner’s pubertal stage, maternal authoritarian parenting style (PSDQ), and parenting stress (NOSI-K).

### Statistical analyses

Associations between descriptive characteristics and maternal verbally aggressive behavior were tested using ANOVA and Chi-square tests. Associations between the different polymorphisms and maternal verbally aggressive behavior were tested using Chi-square tests. Medians of general anxiety, anxiety sensitivity, and both SDQ emotional symptoms at age 5–6 and at age 11–12 according to genotype variant were examined using Mann–Whitney test. As the distribution of the outcome variables was highly skewed, and transformation did not normalize this, we analyzed the rank variables of general anxiety, anxiety sensitivity, and SDQ emotional symptoms scores by means of rank regression [42]. Because the results of rank regression analyses are difficult to interpret, we showed the results of the multivariable linear regression of the outcome variables in Supplementary Tables as well. Potential confounders were selected a priori from Table 2 and included in the regression model using a forced-entry method. We always corrected for sex. In addition, we selected covariates, which were significantly associated with maternal verbally aggressive behavior. We decided to select authoritarian parenting style and parenting stress at age 5–6 only, because we expected those variables to be more relevant in the development of internalizing symptoms than those variables at age 11–12. After initial testing in a univariate model (Crude Model), the following covariates were added to an adjusted model (Model 1): sex, maternal depressive symptoms, pleasure in infant care, and physically aggressive behavior in infancy. Finally, authoritarian parenting style, maternal depression, and parenting stress at the age of 5–6 were added to the fully adjusted model (Model 2). Interaction terms of maternal verbally aggressive behavior and variants of OXTR rs53576 (GG vs. GA/AA) or rs2268498 (TT/TC vs. CC) and maternal verbally aggressive behavior and OXT rs2740210 (CC vs. CA/AA) or rs4813627 (GG vs. GA/AA) were added to the fully adjusted model to investigate gene-environment interaction. In addition, the data were stratified into two groups: presence or absence of the risk alleles and analyzed separately according to the above model. The significance level we used in the study was 5%.

**Table 2:** Characteristics of 969 Dutch women and their children according to maternal verbally aggressive behavior in early infancy (*N* = 103)

	*N* 969	Full sample % or mean (SD)	No maternal verbal aggression to infant *N* = 866 (89.4%)	Maternal verbal aggression to infant *N* = 103 (10.6%)	*p* value
Child characteristics					
Gender (% female)	504	52.0	52.1	51.5	0.91
Birth weight (g)	969	3568 (477)	3561 (479)	3626 (459)	0.19
Gestational age (weeks)	969	39.7 (1.2)	39.7 (1.2)	39.7 (1.1)	0.89
Excessive crying (% yes)	17	1.8	1.6	2.9	0.34
Maternal characteristics					
Parity (% primipara)	535	55.2	54.7	59.2	0.39
Maternal age (years)	969	33.0 (4.0)	33.1 (4,0)	32.5 (4.0)	0.13
Cohabitancy (% living with partner)	899	93.0	92.9	93.1	0.94
Education (years after primary school)	967	10.7 (3.0)	10.8 (2.9)	10.4 (3.0)	0.25
Maternal psychiatric history					
Autistic disorder	968	0.0	0.0	0.0	n.a.
ADHD	967	0.6	0.0	0.7	0.51
Schizophrenia	967	0.1	0.1	0.0	0.89
Bipolar disorder	968	0.5	0.2	2.9	0.01
Depressive disorder	968	15.3	14.1	25.2	0.004
Anxiety disorder	966	6.8	6.0	13.6	0.007
Maternal factors in infancy					
Depressive symptoms	968	7.8 (6.3)	7.4 (6.1)	11.1 (7.1)	< 0.001
Pleasure in infant care	964	5.9 (1.4)	5.8 (1.4)	6.5 (1.7)	< 0.001
Smoking at home (% yes)	37	3.8	4.0	1.9	0.29
Physically aggressive behavior	969	3.0 (0.2)	3.0 (0.1)	3.2 (0.5)	< 0.001
Child age 5–6					
Intact family (% yes)	867	89.6	90.0	85.4	0.15
Authoritarian parenting style	941	4.7 (2.6)	4.7 (2.6)	5.3 (2.8)	0.03
Maternal depressive symptoms	941	0.9 (1.8)	0.8 (1.7)	1.5 (2.4)	< 0.001
Parenting stress	834	11.5 (2.7)	11.4 (2.7)	12.1 (3.0)	0.01
Child age 11–12 years					
Puberty, Tanner stage 3	48	6.9	40 (6.5)	8 (9.9)	0.31
Child age 11–12					
Authoritarian parenting style	709	1.3 (0.2)	1.3 (0.2)	1.4 (0.2)	0.001
Parenting stress	707	24.2 (7.2)	23.9 (7.0)	26.8 (8.7)	0.001
Intact family (% yes)	612	79.3	79.6	76.5	0.291

## Results

### Subjects’ characteristics

The characteristics of both mothers and children are presented in Table 2. Of the 969 included children, 103 (10.6%) had been exposed to maternal verbally aggressive behavior. Compared to mothers of non-exposed children, mothers of exposed children reported more depressive symptoms, less pleasure in infant care, and more physical aggressive behaviour in infancy, and prevalence of bipolar disorder or a history of depressive or anxiety disorder was higher. At the child’s age of 5–6, mothers of exposed children reported more depressive symptoms, more parenting stress, and more often used an authoritarian parenting style, compared to mothers of non-exposed children. At the child’s age of 11–12, mothers of exposed children reported more parenting stress, and more often used an authoritarian parenting style, compared to mothers of non-exposed children.

### Maternal verbally aggressive behavior and child’s OXTR and OXT variants

Maternal verbally aggressive behavior in infancy was neither associated with infant’s OXTR nor OXT variants (Table 3).

**Table 3:** OXTR and OXTP genotype variants according to maternal verbally aggressive behavior in infancy, general anxiety, SDQ emotional symptoms by maternal report at age 5–6, and anxiety sensitivity and SDQ emotional symptoms by self-report at age 11–12

	*N* (%)	No maternal verbally aggressive behavior *N* (%)	Maternal verbally aggressive behavior *N* (%)	General anxiety Age 5–6 Median	SDQ emotional symptoms Age 5–6 Median	Anxiety sensitivity Age 11–12 Median	SDQ emotional symptoms Age 11–12 Median
OXTR rs53576							
GG (wild type)	406 (41.9)	366 (42.3)	40 (38.8)	0.0	1.0	1.8	2.0
GA	459 (47.4)	412 (47.6)	47 (45.6)	0.0	0.0	1.8	1.0
AA	104 (10.7)	88 (10.2)	16 (15.5)	0.0	0.0	1.8	1.0
OXTR rs2268498							
TT (wild type)	287 (29.6)	260 (30)	27 (26.2)	0.0	0.0	1.8	2.0
TC	487 (50.3)	430 (49.7)	57 (55.3)	1.0	1.0	1.8	2.0
CC	195 (20.1)	176 (20.3)	19 (18.4)	0.0	0.0	1.8	1.0
OXTP rs2740210							
CC (wild type)	475 (49.0)	417 (48.2)	58 (56.3)	0.0	0.0	1.8	2.0
CA	404 (41.7)	369 (42.6)	35 (34.0)	1.0	1.0	1.8	2.0
AA	90 (9.3)	80 (9.2)	10 (9.7)	0.0	0.0	1.7	1.0
OXTP rs4813627							
GG (wild type)	275 (28.4)	245 (28.3)	30 (29.1)	0.0	0.0	1.8	2.0
GA	460 (47.5)	411 (47.5)	49 (47.6)	1.0	1.0	1.8	2.0
AA	234 (24.1)	210 (24.2)	24 (23.3)	0.0	0.0	1.8	1.0*

* < 0.05; ** < 0.01; *** < 0.001

### OXTR, OXT variants, and median score of internalizing symptoms at age 5–6 and age 11–12

Median scores of general anxiety, anxiety sensitivity and SDQ emotional symptoms at age 5–6 and age 11–12 are shown in Table 3. Only genotype variant rs4813627 AA was significantly associated with a lower median score of SDQ emotional symptoms at age 11–12 (Mann–Whitney *p* = 0.009).

### Maternal verbally aggressive behavior and internalizing symptoms at age 5–6 and age 11–12

Results from the rank regression showed that maternal verbally aggressive behavior was not associated with general anxiety, neither univariably (Crude model) (*B* = 29.8; 95% CI [(− 22.4; 82.0]), nor multivariable (Model 2) (*B* = 22.5; 95% CI [− 33.3; 78.4]). Maternal verbally aggressive behavior was neither associated with SDQ emotional symptoms at age 5–6, neither univariably (Crude model) (*B* = 0.0; 95% CI [− 52.5; 52.5]), nor multivariable (Model 2) (*B* = − 20.6; 95% CI [− 76.2; 35.0]) nor at age 11–12, neither univariably (Crude model) (*B* = − 2.6; 95% CI [− 51.1; 45.9]), nor multivariable (Model 2) (*B* = − 4.2; 95% CI [− 55.6; 47.2]). Finally, maternal verbally aggressive behavior was not associated with anxiety sensitivity at age 11–12, neither univariably (Crude model) (*B* = 3.3; 95% CI [− 43.7; 50.4]), nor multivariable (Model 2) (*B* = 0.7; 95% CI [− 48.7; 50.1]) (Table S1).

### OXTR and OXT variants, maternal verbally aggressive behavior, and internalizing symptoms at age 5–6

**Fig. 2:**
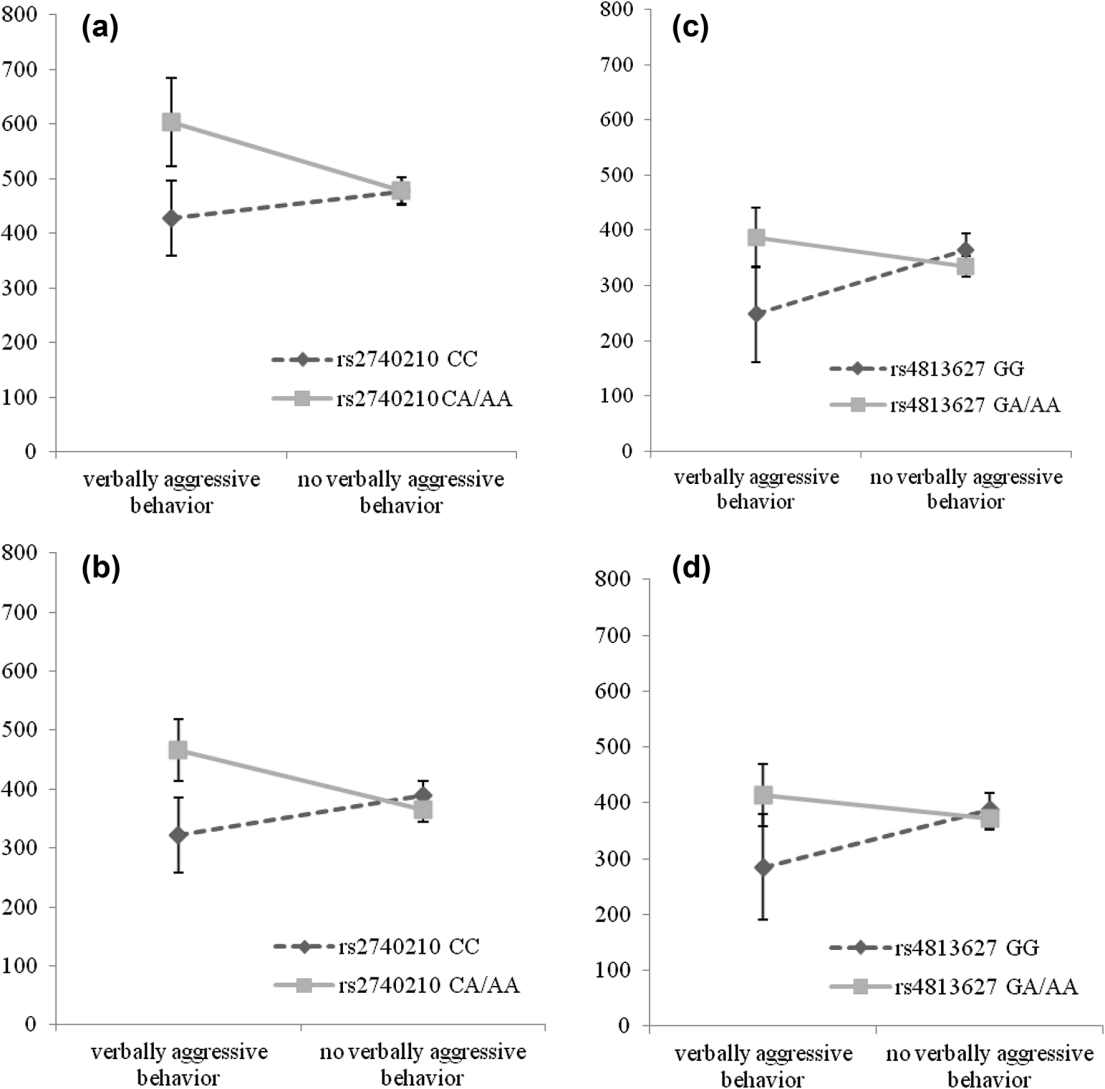
**a** General anxiety at age 5–6 (ranking scores) according to maternal verbally aggressive behavior, stratified by rs2740210. **b** SDQ-emotional symptoms at age 11–12 (ranking variables) according to maternal verbally aggressive behavior, stratified by rs2740210. **c** Anxiety sensitivity at age 11–12 (ranking variables) according to maternal verbally aggressive behavior, stratified by rs4813627. **d** SDQ-emotional symptoms at age 11–12 (ranking variables) according to maternal verbally aggressive behavior, stratified by rs4813627. Oxytocin peptide genotype variants influence internalizing symptoms (ranking variables) after exposure to maternal verbally aggressive behavior in early infancy. Carriers of rs2740210 CA/AA exposed to maternal verbally aggressive behavior in early infancy have increased general anxiety symptoms at age 5–6 and increased Goodman’s Strengths and Difficulties Questionnaire (SDQ) emotional symptoms at age 11–12, compared to carriers of rs2740210 CA/AA unexposed to maternal verbally aggressive behavior in early infancy (interaction terms, respectively, 0.011 and 0.015). Carriers of rs4813627 GG exposed to maternal verbally aggressive behavior in early infancy have decreased anxiety sensitivity and decreased SDQ emotional symptoms at age 11–12, compared to carriers of rs4813627 GG unexposed to maternal verbally aggressive behavior in early infancy (interaction terms, respectively, 0.011 and 0.023)

Results from the rank regression showed that exposed carriers of rs2740210 CA/AA showed increased general anxiety problems at age 5–6 and SDQ emotional symptoms at age 11–12, compared to unexposed carriers of rs2740210 CA/AA (*p* value for interaction *p* = 0.011 and *p* = 0.015, respectively) (Fig. 2a, b). Exposed carriers of rs4813627 GG (the wild type) showed decreased anxiety sensitivity and SDQ-emotional symptoms at age 11–12, compared to unexposed carriers of rs4813627 GG (*p* value for interaction *p* = 0.011 and *p* = 0.023, respectively) (Fig. 2c, d). There was no significant interaction between exposure and rs53576 GG or rs2268498 TT/TC on internalizing outcomes (Table S2).

## Discussion

Our study shows novel evidence, suggesting that OXT polymorphisms might influence the vulnerability or resilience to develop internalizing symptoms in childhood and pre-adolescence, after exposure to maternal verbally aggressive behavior in early infancy. In line with our hypothesis, we found evidence for gene-environment interaction of OXT polymorphisms previously shown to be associated with duration of maternal vocalization [25]. Our results suggest that carrying OXT variant rs2740210 CA/AA increases the risk to develop general anxiety problems at age 5–6 and SDQ-emotional symptoms at age 11–12, after exposure to maternal verbally aggressive behavior. Interestingly, carriers of OXT variant rs4813627 GG are suggested to benefit from maternal verbally aggressive behavior, as these carriers show decreased anxiety sensitivity and SDQ emotional symptoms at age 11–12, after exposure to maternal verbally aggressive behavior, compared to GA/AA carriers. Thus, both wild types of the child’s OXT variants, shown to be associated with longer duration of vocalizing in mothers, seem protective after exposure to maternal verbally aggressive behavior. Although children’s phenotype of these OXT SNPs is unknown until now, we would like to elaborate on our findings in this discussion. Prosody, defined as variations in rhythm, intonation, and pitch, is a feature of mammalian vocalization which communicates emotional charges and affective state [43]. Although speculative, it is possible that carriers of the OXT wild type differ from the risk carriers in seeking communication, in the ability to reorient to (or filter out) salient stimuli and possibly in OXT release. Subsequently, programming effects of the OXT, and communicating systems, might differentially occur. Of note, no main effect was found of maternal verbally aggressive behavior and internalizing symptoms both at age 5–6 and age 11–12, contrarily to other studies on parental verbal aggression in childhood [9, 10, 11]. Importantly, we did not investigate persistent maternal verbally aggressive behavior to the child. On the contrary, we aimed to focus on the single stressor of maternal verbal aggressive behavior in early infancy.

Our null findings on OXTR variants rs53576 and rs2268498 are in contrast with our hypothesis and the studies by McQuaid [17] and Bradley [18], but in line with the study of Tollenaar et al. [19], in which no interaction was shown of neither rs53576 nor rs2268498 and childhood maltreatment by retrospective recall. Interestingly, ample evidence suggests possible psychological resilience in rs53576 GG carriers, compared to A-allele carriers [44, 45], due to their innate higher support seeking, higher levels of optimism, mastery, self-esteem and decreased emotion-focused coping following unsupportive responses, compared to the A-allele carriers. Developing empathic abilities in hypothesized OXTR risk allele carriers could help in time to overcome stressors and induce self-regulation, in a way that G-carriers more than in A-carriers, respectively, TT/TC carriers more than CC carriers, feel able to cope with external demands. Indeed, the developing mastery capacities of the hypothesized OXTR risk allele carriers might explain our null finding on internalizing symptoms, but only in the plausible absence of cumulative maternal abuse in due time. Instead, adolescent carriers of rs53576 GG variant with documented maltreatment histories, on average an accumulation of 2.2 maltreatment subtypes have been shown to have higher levels of internalizing symptoms and perceive lower social support compared to maltreated A-carriers [18]. Second, it is possible that the absence of gene-environment interaction of OXTR variants is partly due to the fact that we could not assess the possible contribution of the developing attachment style. Indeed, in adult rs53576 GG carriers, insecure childhood attachment is associated with higher attachment related anxiety and alexithymia, than in A-allele carriers [46].

### Strengths and limitations

Strengths of this study are the large, population-based, birth cohort with prospective design and extensive data collection from early infancy onwards. General anxiety, anxiety sensitivity, and SDQ-emotional symptoms cover multiple aspects of internalizing symptoms. Since they belong to separate high-risk groups, we excluded preterm births and congenital disorders. We were able to control for a large number of potentially confounding stressors, such as maternal depression, authoritarian parenting style, and parenting stress, as we were specifically interested in the impact of maternal verbally aggressive behavior in early infancy. Importantly, gonadal steroids, which rise in puberty, have a role in the maturation of the oxytocin system [47] and endocrinological changes could co-exist with internalizing symptoms. Nevertheless, in our sample, puberty stage did not differ between the exposed and the non-exposed children. To follow the recommendations of Keller [48] to properly control for potential confounders, we additionally checked whether adding sex × G, sex × E, physical aggression × E, and physical aggression × G to the complete model would change the results. The *p* values for interaction of the SNPs and maternal verbal aggression in association with internalizing symptoms minimally changed and remained significant [rs2740210 × verbal aggression: *p* = 0.002 (instead of 0.011) and *p* = 0.002 (instead of 0.015); rs4813627 × verbal aggression: *p* = 0.015 (instead of 0.011) and *p* = 0.009 (instead of 0.023)]. Possible social desirability in answering the question of speaking angrily to the infant could lead to underestimation of the true frequency of maternal verbally aggressive behavior. However, self-report is the best way to measure this as continuous observation of the mother–infant dyad is not feasible.

Selective follow-up was present as in most cohort studies. However, as maternal verbally aggressive behavior did not differ between responders and non-responders, respectively, 10.6% and 9.2% (*p* = 0.177), possible selection bias is estimated to be limited. Furthermore, the genotype variants did not vary across the exposed and non-exposed children. Therefore, no population stratification was present. Since parents have been shown to underestimate child worry and anxiety and overestimate optimism, compared to child self-report in 4–11-year-old children [49], we cannot rule out the possibility that internalizing symptoms at age 5–6 could have been underestimated. Although we assessed internalizing symptoms at age 5–6 by maternal report only, we did assess internalizing symptoms at age 11–12 by self-report. Unfortunately, paternal report was not collected. Data on trauma in early childhood were limited to maternal aggressive behavior to the infant at the age of 3 months. The distinction between temporary, frequent, or persistent maternal aggressive behavior to the infant, thus creating chronic stress in the first years, could not be made based on our data. We expect that experience of simultaneous other adverse events will increase the gene–environment interaction effect.

Although the study population was large, the numbers of subjects in various groups in the studied interactions seem small (ranging from 19–44), nonetheless, comparable to other gene-environment studies. We aimed to replicate the results in an independent cohort, but to the best of our knowledge, no other cohort study exists in which maternal verbally aggressive behaviour at the age of 3 months was assessed. Nevertheless, this is an exploratory analysis and our findings on gene-environment interaction by OXT polymorphisms should be replicated in independent samples. Furthermore, gene–environment interaction of OXTR or OXT polymorphisms could differ between boys and girls. Indeed, evidence of sex differences exists in brain structure, function, and neurotransmission [50] as well as stress reactivity [51]. Unfortunately, our study was underpowered for sex-stratified interaction analyses.

Interestingly, it might be possible that the environmental stressor of verbal aggression differs from other types of maltreatment (i.e., neglect, physical abuse, and sexual abuse) in interaction with OXTR or OXT polymorphisms, as such has been shown in the serotonin transporter gene [52]. In this study of Fisher et al. (2013), gene–environment interactions were found only when maltreatment was analyzed in accumulation, or when sexual abuse or physical neglect was analyzed separately. A separate study on gene–environment interaction for each type of maltreatment in early infancy might show different results. Importantly, we tested the effect of gene–environment interaction on internalizing symptoms along the internalizing symptoms continuum. We were unable to examine a differential effect on prevalence of mood or anxiety disorder because of our healthy population sample. Future studies should incorporate clinical outcomes, as well as sex differences in larger birth cohort samples and continue into adolescence and adulthood. Eventually, studies of possible genetic overlapping functions are warranted to further explore the relevance of these promising genetic markers.

This study uniquely contributes to the field. Infant’s genetic variation of OXT polymorphisms associated with vocalizing is shown to be a significant factor of vulnerability or resilience in developing internalizing symptoms after exposure to maternal verbally aggressive behavior in early infancy. Common psychiatric disorders as mood and anxiety disorders have been recognized as continuous phenotypes in the population. Therefore, aetiopathogenic mechanisms should be detectable across a wide range of subclinical and clinical phenotypic variants in non-clinical samples such as the ABCD cohort.

## Electronic supplementary material

Below is the link to the electronic supplementary material.

Supplementary file1 (DOC 88 kb)

## References

[1] Seltzer LJ, Ziegler TE, Pollak SD (2010). Social vocalizations can release oxytocin in humans. Proc Biol Sci.

[2] Feldman R (2017). The Neurobiology of human attachments. Trends Cogn Sci.

[3] Haber SN, Gottfried JA (2011). Neuroanatomy of reward: a view from the ventral striatum. Neurobiology of sensation and reward.

[4] Robinson JL, Laird AR, Glahn DC, Blangero J, Sanghera MK, Pessoa L (2012). The functional connectivity of the human caudate: an application of meta-analytic connectivity modeling with behavioral filtering. Neuroimage.

[5] Wise RA (2004). Dopamine, learning and motivation. Nat Rev Neurosci.

[6] Bromberg-Martin ES, Matsumoto M, Hikosaka O (2010). Dopamine in motivational control: rewarding, aversive, and alerting. Neuron.

[7] Huttenlocher PR, de Courten C (1987). The development of synapses in striate cortex of man. Hum Neurobiol.

[8] Volman SF, Lammel S, Margolis EB, Kim Y, Richard JM, Roitman MF (2013). New insights into the specificity and plasticity of reward and aversion encoding in the mesolimbic system. J Neurosci.

[9] Carr CP, Martins CM, Stingel AM, Lemgruber VB, Juruena MF (2013). The role of early life stress in adult psychiatric disorders: a systematic review according to childhood trauma subtypes. J Nerv Ment Dis.

[10] Donovan KL, Brassard MR (2011). Trajectories of maternal verbal aggression across the middle school years (age 9–12): associations with negative view of self and social problems. Child Abuse Negl.

[11] Scher CD, Stein MB (2003). Developmental antecedents of anxiety sensitivity. J Anxiety Disord.

[12] Bounoua N, Felton JF, Long K, Stadnik RD, Loya JM, MacPherson L (2015). Childhood emotional abuse and borderline personality features: The role of anxiety sensitivity among adolescents. Personal Ment Health.

[13] Sachs-Ericsson N, Verona E, Joiner T, Preacher KJ (2006). Parental verbal abuse and the mediating role of self-criticism in adult internalizing disorders. J Affect Disord.

[14] Osório C, Probert T, Jones E, Young AH, Robbins I (2017). Adapting to stress: understanding the neurobiology of resilience. Behav Med.

[15] Gong P, Fan H, Liu J, Yang X, Zhang K, Zhou X (2017). Revisiting the impact of OXTR rs53576 on empathy: a population-based study and a meta-analysis. Psychoneuroendocrinology.

[16] Dannlowski U, Kugel H, Grotegerd D, Redlich R, Opel N, Dohm K (2016). Disadvantage of social sensitivity: interaction of oxytocin receptor genotype and child maltreatment on brain structure. Biol Psychiatry.

[17] McQuaid RJ, McInnis OA, Stead JD, Matheson K, Anisman H (2013). A paradoxical association of an oxytocin receptor gene polymorphism: early-life adversity and vulnerability to depression. Front Neurosci.

[18] Hostinar CE, Cicchetti D, Rogosch FA (2014). Oxytocin receptor gene polymorphism, perceived social support, and psychological symptoms in maltreated adolescents. Dev Psychopathol.

[19] Andreou D, Comasco E, Åslund C, Nilsson KW, Hodgins S (2018). Maltreatment, the oxytocin receptor gene, and conduct problems among male and female teenagers. Front Hum Neurosci.

[20] Bradley B, Westen D, Mercer KB, Binder EB, Jovanovic T, Crain D (2011). Association between childhood maltreatment and adult emotional dysregulation in a low-income, urban, African American sample: moderation by oxytocin receptor gene. Dev Psychopathol.

[21] Tollenaar MS, Molendijk ML, Penninx BW, Milaneschi Y, Antypa N (2017). The association of childhood maltreatment with depression and anxiety is not moderated by the oxytocin receptor gene. Eur Arch Psychiatry Clin Neurosci.

[22] Melchers M, Montag C, Markett S, Reuter M (2013). Relationship between oxytocin receptor genotype and recognition of facial emotion. Behav Neurosci.

[23] Melchers M, Montag C, Markett S, Niazy N, Groß-Bölting J, Zimmermann J (2017). The OXTR gene, implicit learning and social processing: Does empathy evolve from perceptual skills for details?. Behav Brain Res.

[24] Christ CC, Carlo G, Stoltenberg SF (2016). Oxytocin receptor (OXTR) single nucleotide polymorphisms indirectly predict prosocial behavior through perspective taking and empathic concern. J Pers.

[25] Mileva-Seitz V, Steiner M, Atkinson L, Meaney MJ, Levitan R, Kennedy JL (2013). Interaction between oxytocin genotypes and early experience predicts quality of mothering and postpartum mood. PLoS One.

[26] Van Eijsden E, Vrijkotte TG, Gemke RJBJ, van der Wal MF (2011). Cohort profile: the Amsterdam Born Children and their Development (ABCD) study. Int J Epidemiol.

[27] Smarius LJCA, Strieder TGA, Doreleijers TAH, Vrijkotte TGM, de Rooij SR (2018). Maternal verbally aggressive behavior in early infancy is associated with blood pressure at age 5–6. J Dev Orig Health Dis.

[28] Van Widenfelt BM, Goedhart AW, Treffers PD, Goodman R (2003). Dutch version of the Strengths and Difficulties Questionnaire (SDQ). Eur Child Adolesc Psychiatry.

[29] Edwards SL, Rapee RM, Kennedy SJ, Spence SH (2010). The assessment of anxiety symptoms in preschool-aged children: the revised Preschool Anxiety Scale. J Clin Child Adolesc Psychol.

[30] Woicik PA, Conrod PJ, Phil RO, Stewart SH, Dongier M (1999) The drug abuse subtyping scale: a revised instrument for identifying motivational profiles for substance abuse. In: Poster presented at the 22nd annual meeting of the research society on alcoholism, Santa Barbara

[31] Pistis G, Porcu E, Vrieze SI, Sidore C, Steri M, Danjou F (2015). Rare variant genotype imputation with thousands of study-specific whole-genome sequences: implications for cost-effective study designs. Eur J Hum Genet.

[32] Stringer S, Cerrone KC, van den Brink W, van den Berg JF, Denys D, Kahn RS (2015). A guide on gene prioritization in studies of psychiatric disorders. Int J Methods Psychiatr Res.

[33] Sharma S, Powers A, Bradley B, Ressler KJ (2016). Gene × environment determinants of stress- and anxiety-related disorders. Annu Rev Psychol.

[34] Stenzel SL, Ahn J, Boonstra PS, Gruber SB, Mukherjee B (2015). The impact of exposure-biased sampling designs on detection of gene-environment interactions in case-control studies with potential exposure misclassification. Eur J Epidemiol.

[35] Kraft P, Aschard H (2015). Finding the missing gene-environment interactions. Eur J Epidemiol.

[36] Smarius LJ, Strieder TG, Loomans EM, Doreleijers TA, Vrijkotte TG, Gemke RJ (2017). Excessive infant crying doubles the risk of mood and behavioral problems at age 5: evidence for mediation by maternal characteristics. Eur Child Adolesc Psychiatry.

[37] Knight RG, Williams S, McGee R, Olaman S (1997). Psychometric properties of the Centre for Epidemiologic Studies Depression Scale (CES-D) in a sample of women in middle life. Behav Res Ther.

[38] Robinson CC, Mandleco B, Olsen SF, Hart CH, Perlmutter BF, Touliatos J, Holden GW (2001). The Parenting Styles and Dimensions Questionnaire (PSDQ). Handbook of family measurement techniques.

[39] Wolford SN, Cooper AN, McWey LM (2018). Maternal depression, maltreatment history, and child outcomes: the role of harsh parenting. Am J Orthopsychiatry.

[40] De Brock AJLL, Vermulst AA, Gerris JRM, Abidin RR (1992) Nijmeegse Ouderlijke Stress Index (NOSI). Handleiding experimentele versie [NOSI-Nijmegen Parenting Stress Index, Manual experimental version]. Lisse, Swets en Zeitlinger

[41] Henry JD, Crawford JR (2005). The short-form version of the Depression Anxiety Stress Scales (DASS-21): construct validity and normative data in a large non-clinical sample. Br J Clin Psychol.

[42] Chen T, Tang W, Lu Y, Tu X (2014). Rank regression: an alternative regression approach for data with outliers. Shanghai Arch Psychiatry.

[43] Saint-Georges C, Chetouani M, Cassel R, Apicella F, Mahdhaoui A, Muratori F (2013). Motherese in interaction: at the cross-road of emotion and cognition? (A systematic review). PLoS One.

[44] Saphire-Bernstein S, Way BM, Kim HS, Sherman DK, Taylor SE (2011). Oxytocin receptor gene (OXTR) is related to psychological resources. Proc Natl Acad Sci USA.

[45] McInnis OA, McQuaid RJ, Matheson K, Anisman H (2015). The moderating role of an oxytocin receptor gene polymorphism in the relation between unsupportive social interactions and coping profiles: implications for depression. Front Psychol.

[46] Schneider-Hassloff H, Straube B, Jansen A, Nuscheler B, Wemken G, Witt SH (2016). Oxytocin receptor polymorphism and childhood social experiences shape adult personality, brain structure and neural correlates of mentalizing. Neuroimage.

[47] Chibbar R, Toma JG, Mitchell BF, Miller FD (1990). Regulation of neural oxytocin gene expression by gonadal steroids in pubertal rats. Mol Endocrinol.

[48] Keller MC (2014). Gene × environment interaction studies have not properly controlled for potential confounders: the problem and the (simple) solution. Biol Psychiatry.

[49] Lagattuta KH, Sayfan L, Bamford C (2012). Do you know how I feel? Parents underestimate worry and overestimate optimism compared to child self-report. J Exp Child Psychol.

[50] Cosgrove KP, Mazure CM, Staley JK (2007). Evolving knowledge of sex differences in brain structure, function, and chemistry. Biol Psychiatry.

[51] Solomon MB, Herman JP (2009). Sex differences in psychopathology: of gonads, adrenals and mental illness. Physiol Behav.

[52] Fisher HL, Cohen-Woods S, Hosang GM, Korszun A, Owen M, Craddock N (2013). Interaction between specific forms of childhood maltreatment and the serotonin transporter gene (5-HTT) in recurrent depressive disorder. J Affect Disord.

