# Supplementary material for: Maternal verbal aggression in early infancy and child’s internalizing symptoms: interaction by common oxytocin polymorphisms
Source: Eur Arch Psychiatry Clin Neurosci. 2019 May 7;270(5):541–51. doi: 10.1007/s00406-019-01013-0 (PMC7332476; doi:10.1007/s00406-019-01013-0)
Supplement: Supplementary file 1 — Supplementary file1 (DOC 88 kb) [file 406_2019_1013_MOESM1_ESM.doc]

**Table S1** *Difference in internalizing symptoms (maternal report at age 5-6 and self-report at age 11-12 years) between children exposed to maternal verbally aggressive behavior in infancy and non-exposed children*

|  | Maternal verbal aggressive behavior in infancy: No  Mean (SD) | Maternal verbal aggressive behavior in infancy: Yes  Mean (SD) | Difference  (95%CI) | Model 1  Adjusted difference (95%CI) | Model 2  Adjusted difference (95%CI) |  |
| --- | --- | --- | --- | --- | --- | --- |
| *Age 5-6*  General anxiety | 0.8 (1.1) | 1.0 (1.5) | 0.3 (0.0,0.5)* | 0.2 (-0.0,0.5) | 0.2 (-0.0,0.5) |  |
| General anxiety rank | 481.8 (252.2) | 511.7 (297.1) | 29.8 (-22.2,82.0) | 25.0 (-30.7,80.6) | 22.5 (-33.3,78.4) |  |
| SDQ emotional symptoms  SDQ emotional symptoms rank  *Age 11-12* | 0.8 (1.1)  485.0 (255.3) | 0.9 (1.4)  485.0 (268.8) | 0.1 (-0.2,0.3)  0.0 (-52.5,52.5) | 0.1 (-0.2,0.3)  -12.8 (-68.7,43.0) | 0.0 (-0.2,0.3)  -20.6 (-76.2,35.0) |  |
| Anxiety sensitivity  Anxiety sensitivity rank  SDQ emotional symptoms  SDQ emotional symptoms rank | 1.9 (0.6)  344.6 (197.3)  1.9 (1.8)  375.8 (211.9) | 1.9 (0.6)  348.0 (204.0)  1.9 (1.7)  373.2 (215.1) | -0.0 (-0.2,0.1)  3.3 (-43.7,50.4)  -0.1 (-0.5,0.4)  -2.6 (-51.1,45.9) | -0.0 (-0.2,0.1)  4.5 (-44.6,53.6)  -0.1 (-0.5,0.4)  -0.5 (-51.8,50.8) | -0.0 (-0.2,0.1)  0.7 (-48.7,50.1)  -0.1 (-0.5,0.4)  -4.2 (-55.6,47.2) |  |

SDQ= Dutch translation of Goodman’s Strenghts and Difficulties Questionnaire

Model 1: adjusted for sex, maternal depressive symptoms, pleasure in infant care, maternal physical aggression in infancy; Model 2: Model 1, additionally adjusted for authoritarian parenting style, maternal depressive symptoms and parenting stress (NOSI-K) at the child’s age of 5-6. *P<0.05

**Table S2** *Difference in internalizing symptoms (maternal report at age 5-6 years and self-report at age 11-12 years) between children*

*exposed to maternal verbally aggressive behavior in infancy and non-exposed children, stratified for presence or absence of oxytocin genetic risk variants*

|  | **Oxytocin Polymorphism**  **rs53576** | Maternal verbally aggressive behavior in infancy  Mean (SD)  No Yes | | Difference  (95%CI) | Model 1  Infancy  Adjusted difference (95%CI) | Model 2  Age 5-6  Adjusted difference (95%CI) | Inter-action  p-value^*^ |  |
| --- | --- | --- | --- | --- | --- | --- | --- | --- |
| *Age 5-6*  General anxiety  General anxiety rank  SDQ emotional symptoms  SDQ emotional symptoms rank | GG  GA/AA  GG  GA/AA  GG  GA/AA  GG  GA/AA | 0.8 ± 1.0  0.8 ± 1.1  482.9 ± 250.1  480.9 ± 254.0  0.8 ± 1.0  0.8 ± 1.2  489.2 ± 252.3  481.9 ± 257.6 | 1.2 ± 1.9  0.9 ± 1.2  519.6 ± 284.0  506.6 ± 278.1  1.1 ± 1.8  0.8 ± 1.1  496.2 ± 279.6  477.9 ± 263.7 | 0.47 (0.10;0.84)*  0.14 (-0.16;0.43)  36.6 (-46.4;119.6)  25.6 (-41.8;93.1)  0.27 (-0.09;0.64)  -0.02 (-0.33;0.28)  6.9 (-76.5;90.4)  -4.0 (-71.8;63.9) | 0.48 (0.10;0.87)*  0.07 (-0.25;0.38)  37.0 (-50.2;124.1)  19.8 (-53.4;92.9)  0.28 (-0.09;0.66)  -0.07 (-0.41;0.26)  8.8 (-77.9;95.5)  22.1 (-95.6;51.4) | 0.48 (0.09;0.87)*  0.04 (-0.28;0.36)  34.6 (-53.4;122.5)  13.9 (-59.3;87.1)  0.25 (-0.31;0.63)  -0.13 (-0.46;0.20)  1.0 (-86.3;88.4)  -33.2 (-105.7;39.4) | 0.626  0.838  0.165  0.659 |  |
| *Age 11-12*  Anxiety sensitivity  Anxiety sensitivity rank  SDQ emotional symptoms  SDQ emotional symptoms rank | GG  GA/AA  GG  GA/AA    GG  GA/AA    GG  GA/AA | 1.9± 0.6  1.9± 0.6  352.9 ± 196.2  338.8 ± 198.2  2.0± 1.9  1.8± 1.7  392.8 ± 213.6  363.9 ± 210.1 | 1.9± 0.6  1.9± 0.6  359.0 ± 211.1  339.2 ± 200.3  2.1± 1.9  1.6± 1.5  401.1± 220.4  353.8 ± 211.5 | -0.01 (-0.22;0.20)  -0.03 (-0.21;0.16)  6.1(-65.1;77.3)  0.4 (-62.5;63.3)  0.07 (-0.63;0.76)  -0.14 (-0.66;0.37)  8.4 (-68.3;85.1)  -10.1 (-72.7;52.5) | 0.01 (-0.21;0.23)  -0.03 (-0.23;0.16)  14.6 (-59.4;88.6)  -1.2 (-67.4;65.0)  0.14(-0.59;0.86)  -0.22(-0.77;0.34)  19.4 (-60.3;99.0)  -19.7 (-86.9;47.4) | -0.01 (-0.23;0.20)  -0.05 (-0.24;0.15)  9.4 (-65.3;84.1)  -7.2 (-74.0;59.6)  0.13(-0.61;0.86)  -0.23 (-0.78;0.33)  16.8 (-64.0;97.6)  -19.9 (-87.2;47.4) | 0.754  0.751  0.654  0.798 |  |
|  | **Oxytocin Polymorphism**  **rs 2268498** | Maternal verbally aggressive behavior in infancy  Mean (SD)  No Yes | | Difference  (95%CI) | Model 1  Infancy  Adjusted difference (95%CI) | Model 2  Age 5-6  Adjusted difference (95%CI) | Inter-action  P-value^*^ |  |
| *Age 5-6*  General anxiety | TT/TC  CC | 0.8 ± 1.0  0.8 ± 1.1 | 1.1 ± 1.6  0.8 ± 1.3 | 0.30 (0.06;0.56)*  0.09 (-0.46;0.63) | 0.33 (0.06;0.56)*  -0.27 (-0.84;0.30) | 0.33 (0.06;0.60)*  -0.32 (-0.90;0.26) | 0.202 |  |
| General anxiety rank  SDQ emotional symptoms 5-6  SDQ emotional symptoms rank  *Age 11-12*  Anxiety sensitivity  Anxiety sensitivity rank  SDQ emotional symptoms  SDQ emotional symptoms rank | TT/TC  CC  TT/TC  CC    TT/TC  CC    TT/TC  CC  TT/TC  CC  TT/TC  CC  TT/TC  CC | 484.4 ± 251.6  471.6 ± 254.6  0.8 ± 1.0  0.8 ± 1.1  489.5 ± 257.2  467.2 ± 247.3  1.9 ±0.6  1.9 ±0.5  347.8 ± 200.3  331.9 ±185.4  2.0 ± 1.9  1.6 ± 1.7  384.2 ±211.2  343.6 ±212.2 | 520.2 ± 280.3  473.8 ± 277.7  0.9 ± 1.5  0.8 ± 1.1  487.7 ± 272.3  473.0 ± 259.0  1.9 ±0.6  1.9 ±0.6  351.4 ± 201.8  332.6 ± 220.8  2.0 ± 1.7  1.2 ± 1.5  394.1 ±214.8  291.1 ±202.2 | 35.8 (-22.1;93.6)  2.3 (-120.1;124.6)  0.10 (-0.16;0.36)  0.04 (-0.49;0.57)    -1.8 (-60.5;56.9)  5.8 (-112.5;124.2)  -0.03 (-0.19;0.13)  0.03 (-0.26;0.33)  3.5 (-49.3;56.2)  0.8 (-104.6;106.1)  0.03 (-0.45;0.50)  -0.40 (-1.23;0.44)  9.9 (-44.3;64.2)  -51.5 (-158.7;55.7) | 44.7 (-17.0;106.5)  -68.2 (-197.9;61.5)  0.09 (-0.19;0.36)  -0.15 (-0.72;0.43)    -12.8 (-75.3;49.8)  -37.8 (-165.2;89.5)  -0.02 (-0.19;0.14)  -0.04 (-0.34;0.26)  6.2 (-49.5;62.0)  -25.2 (-133.1;82.8)  0.06 (-0.45;0.56)  -0.43 (-1.32;0.47)  16.8 (-40.7;74.2)  -60.2 (-174.1;53.7) | 43.6 (-18.4;105.5)  -74.3 (-206.2;57.5)  0.06 (-0.22;0.34)  -0.27 (-0.85;0.31)    -18.4 (-80.7;43.9)  -64.4 (-193.5;64.8)  -0.03 (-0.20;0.13)  -0.03 (-0.35;0.28)  3.5 (-52.6;59.5)  -18.4 (-130.2;93.4)  0.03 (-0.48;0.53)  -0.27 (-1.18;0.64)  12.6 (-44.9;70.0)  -42.0 (-158.6;74.6) | 0.420  0.543  0.613  0.758  0.466  0.545  0.386 |  |
|  | **Oxytocin Polymorphism**  **rs 2740210** | Maternal verbally aggressive behavior in infancy  Mean (SD)  No Yes | | Difference  (95%CI) | Model 1  Infancy  Adjusted difference (95%CI) | Model 2  Age 5-6  Adjusted difference (95%CI) | Inter-action  P-value^*^ |  |
| *Age 5-6* |  |  |  |  |  |  |  |  |
| General anxiety | CC  CA/AA | 0.7 ± 1.0  0.8 ± 1.0 | 0.6 ± 0.9  1.6 ± 1.9 | -0.11 (-0.39;0.18)  0.76 (0.40;1.12)*** | -0.17 (-0.48;0.14)  0.80 (0.42;1.18)*** | -0.18 (-0.49;0.13)  0.79 (0.41;1.17)*** | <0.001 |  |
| General anxiety rank    SDQ emotional symptoms 5-6  SDQ emotional symptoms rank  *Age 11-12*  Anxiety sensitivity    Anxiety sensitivity rank  SDQ emotional symptoms  SDQ emotional symptoms rank | CC  CA/AA  CC  CA/AA  CC  CA/AA    CC  CA/AA    CC  CA/AA    CC  CA/AA    CC  CA/AA | 474.4 ± 248.3  487.7 ± 255.8  0.8 ± 1.1  0.9 ± 1.1  473.7 ± 256.5  364.8 ± 205.6  1.9 ±0.6  1.9 ±0.6  355.8 ± 197.3  334.1 ± 197.1  2.0±2.0  1.8±1.7  387.7 ± 218.1  364.8 ± 205.6 | 448.2 ± 250.6  593.4 ± 295.1*  0.6 ± 0.9  1.3 ± 1.8  423.8 ± 245.2  434.5 ± 213.6  1.8 ±0.5  2.1 ±0.6  312.7 ± 195.8  397.6 ± 208.0  1.5±1.6  2.3±1.8  328.5 ± 207.1  434.5 ± 213.6 | -27.2 (-95.7;41.2)  105.7 (25.9;185.4)*  -0.20 (-0.50;0.10)  0.48 (0.12;0.85)**  -49.9 (-120.2;20.3)  68.4 (-10.3;147.2)  -0.16 (-0.34;0.02)  0.17 (-0.05;0.38)  -43.2 (-105.2;18.9)  63.5 (-8.8;135.8)  -0.57 (-1.16;0.02)  0.57 (-0.01;1.16)  -59.1 (-125.1;6.8)  69.7 (-2.3;141.6) | -45.2 (-118.7;28.3)  125.3 (40.8;209.9)**  -0.22 (-0.54;0.10)  0.48 (0.09:0.87)*  -51.3 (-126.4;23.8)  52.6 (-31.8;137.1)  -0.13 (-0.31;0.06)  0.14 (-0.10;0.37)  -31.0 (-94.6;32.7)  53.0 (-24.6;130.7)  -0.65 (-1.28;-0.02)*  0.66 (0.05;1.28)*  -69.7 (-139.7;0.2)  86.3 (10.4;162.1)* | -48.4 (-122.0;25.2)  124.0 (38.4;209.5)**  -0.25 (-0.57;0.08)  0.43 (0.04;0.82)*  -57.2 (-131.9;17.6)  39.3 (-45.3;124.0)  -0.13 (-0.32;0.05)  0.13 (-0.10;0.37)  -33.3 (-97.3;30.8)  54.2 (-25.4;133.7)  -0.64(-1.27;-0.01)*  0.64 (0.02;1.27)*  -67.2 (-136.9;2.5)  80.9 (4.2;157.5)* | 0.011  0.007  0.059  0.049  0.053  0.011  0.015 |  |
|  | **Oxytocin Polymorphism**  **rs 4813627** | Maternal verbally aggressive behavior in infancy  Mean (SD)  No Yes | | Difference  (95%CI) | Model 1  Infancy  Adjusted difference (95%CI) | Model 2  Age 5-6  Adjusted difference (95%CI) | Inter-action  P-value^*^ |  |
| *Age 5-6*  General anxiety | GG  GA/AA | 0.8 ± 1.1  0.8 ± 1.1 | 0.8 ± 1.2  1.2 ± 1.6 | -0.01 (-0.43;0.40)  0.38 (0.11;0.66)** | -0.06 (-0.49;0.37)  0.37 (0.08;0.67)* | -0.09 (-0.50;0.36)  0.37 (0.08;0.67)* | 0.152 |  |
| General anxiety rank  SDQ emotional symptoms  SDQ emotional symptoms rank  *Age 11-12*  Anxiety sensitivity  Anxiety sensitivity rank  SDQ emotional symptoms  SDQ emotional symptoms rank | GG  GA/AA  GG  GA/AA  GG  GA/AA    GG  GA/AA  GG  GA/AA  GG  GA/AA  GG  GA/AA | 483.7 ± 253.6  481.1 ± 251.8  0.8 ± 1.1  0.8 ± 1.1  491.9 ± 257.7  482.3 ± 254.5  2.0± 0.6  1.9± 0.6  361.1± 199.3  338.2 ± 196.4  2.1± 2.1  1.9± 1.7  384.5 ± 223.3  372.4 ± 207.4 | 450.7 ± 273.8  536.7 ± 279.2  0.7± 1.2  1.0 ± 1.5  415.9 ± 269.4  513.4 ± 265.1  1.7± 0.5  2.0± 0.6  263.4 ± 207.8*  375.7 ± 196.7  1.3± 1.6  2.1± 1.7  289.9 ± 203.2  403.3 ± 212.9 | -32.9 (-130.4;64.5)  55.6 (-6.3;117.5)  -0.18 (0.12;0.85)  0.20 (-0.08;0.48)  -76.0 (-174.6;22.6)  31.2 (-30.9;93.3)  -0.31 (-0.59;-0.03)*  0.08 (-0.08;0.24)  -97.7 (-193.2;-2.3)*  37.5 (-16.4;91.4)  -0.80 (-1.69;0.10)  0.21 (-0.25;0.68)  -94.6 (-192.9;3.8)  30.9 (-24.7;86.4) | -47.5 (-148.5;53.4)  60.9 (-5.9;127.7)  -0.14 (-0.58;0.30)  0.14 (-0.16;0.44)  -68.6 (-171.0;33.7)  10.5 (-56.2;77.3)  -0.34 (-0.62;-0.06)*  0.11 (-0.06;0.28)  -109.2 (-204.4;-13.9)*  48.3 (-9.2;105.8)  -0.86 (-1.79;0.06)  0.29 (-0.21;0.79)  -105.9 (-207.4; -4.4)*  44.9 (-14.4;104.2) | -49.8 (-150.1;50.5)  62.9 (-4.4;130.2)  -0.15 (-0.59;0.29)  0.11 (-0.20;0.41)  -71.9 (-172.5;28.7)  3.9 (-63.1;70.9)  -0.36 (-0.64;-0.09)*  0.11 (-0.06;0.28)  -116.6 (-210.9;-22.3)*  50.6 (-7.5;108.7)  -0.84 (-1.75;0.07)  0.28 (-0.22;0.79)  -101.6 (-200.9;-2.3)*  42.9 (-16.8;102.5) | 0.134  0.189  0.108  0.013  0.011  0.032  0.023 |  |

SDQ= Dutch translation of Goodman’s Strengths and Difficulties Questionnaire

Model 1: adjusted for sex, maternal depressive symptoms, pleasure in infant care,

maternal physical aggression in infancy; Model 2: Model 1, additionally adjusted for authoritarian parenting style, maternal depression and parenting stress

at the child’s age of 5-6.

*P <0.05; **<0.01; ***<0.001

^*^Interaction between maternal verbally aggressive behavior in infancy and oxytocin polymorphism
